# Prediction of Steps in the Evolution of Variola Virus Host Range

**DOI:** 10.1371/journal.pone.0091520

**Published:** 2014-03-13

**Authors:** Chad Smithson, Alex Purdy, Adrian J. Verster, Chris Upton

**Affiliations:** 1 Department of Biochemistry and Microbiology, University of Victoria, Victoria, British Columbia, Canada; 2 Department of Molecular Genetics, University of Toronto, Toronto, Ontario, Canada; University of Texas HSC at San Antonio, United States of America

## Abstract

Variola virus, the agent of smallpox, has a severely restricted host range (humans) but a devastatingly high mortality rate. Although smallpox has been eradicated by a World Health Organization vaccination program, knowledge of the evolutionary processes by which human super-pathogens such as variola virus arise is important. By analyzing the evolution of variola and other closely related poxviruses at the level of single nucleotide polymorphisms we detected a hotspot of genome variation within the smallpox ortholog of the vaccinia virus O1L gene, which is known to be necessary for efficient replication of vaccinia virus in human cells. These mutations in the variola virus ortholog and the subsequent loss of the functional gene from camelpox virus and taterapox virus, the two closest relatives of variola virus, strongly suggest that changes within this region of the genome may have played a key role in the switch to humans as a host for the ancestral virus and the subsequent host-range restriction that must have occurred to create the phenotype exhibited by smallpox.

## Introduction

The taxonomic family *Poxviridae* consists of a very diverse set of viruses that share the ability to replicate in the cytoplasm of host cells and a linear dsDNA genome structure. This diversity manifests in a variety of ways: 1) genome size ranges from 134–360 kbp; 2) G+C genome composition ranges from 35–78%; 3) host range for the family as a whole is extremely wide (mammals, birds, reptiles and insects); 4) host range for a particular virus maybe broad (e.g. cowpox virus (CPXV)) or narrow (e.g. smallpox, variola virus (VARV)); 5) infection may be acute (e.g. most poxviruses) or persistent (e.g. molluscum contagiosum virus (MCV)).

Without a traditional fossil record, it is difficult to understand the evolutionary pathways of viruses. However, the availability of new host organisms, through evolution or population growth, can play a pivotal role in the evolution of new virus species. Measles virus is believed to have evolved from a rinderpest virus 1000–1500 years ago [1] and it has been calculated that smallpox evolved approximately 3,500 years ago [2], [3]; since both produce acute infections and long-lasting immunity, their evolution and persistence has been linked to availability of suitably sized host populations. Understanding the past evolution of viruses is of interest because, by outlining the mechanisms by which the process occurs, it may help us predict how viruses might evolve in the future and, in particular, how new viral pathogens could emerge. Within the orthopoxviruses, the two groups of viruses currently described as CPXV are thought to be ancestral-like because they have the largest genomes, containing the entire gene set for this family. CPXVs also have the broadest host range, primarily rodents, and it has been suggested that the evolution of the orthopoxviruses has been a gradual restriction of host range with coincidental loss of genes [4]. However, the corollary that all of the lost genes are host-range determinants, themselves, is unlikely to be true; rather, they simply provide a selectable function in a particular host.

One of the most interesting questions in the evolution of the orthopoxviruses is the origin of smallpox, which, over the centuries, may have killed as many as 300 million people; the restricted host range (currently, only humans) and disease severity (up to 30% lethality) are two of the most salient features of this virus [5], [6]. The very high mortality rate is reminiscent of another orthopoxvirus, ectromelia virus (ECTV) [7], [8], and myxoma virus (MYXV) [9], [10], a leporipoxvirus, both of which have host-dependent mortality rates that may approach 100% in susceptible (non-natural) hosts, mice and European rabbits, respectively. It is notable that both of these viruses cause greatly increased lethality when introduced into a susceptible host in the absence of any immediate genetic changes to the viruses, although the subsequent attenuation of MYXV has been well documented after the deliberate virus release in Australia [11], [12]. Thus, the simplest scenario for the generation of smallpox is a host switch, when the human population was of a sufficient size and organization, followed by gene-loss.

Various phylogenetic analyses indicate that VARV, camelpox virus (CMLV) and taterapox virus (TATV) are sole members of an orthopoxvirus clade; these methods [13], [14] assume simple linear evolutionary pathways between the organisms and the resulting trees are an average or consensus of the information. However, with large sequences, tree branches can still receive high support even though small regions of the sequences may conflict with the tree, and since the researcher is placed at a distance from the raw data, such details are not usually apparent. Although some proteins in the terminal regions have been described as being under positive selection [15] and the VARV inhibitor of complement (SPICE; [16], [17]) has been shown to have adaptions to human complement, to date, the process of smallpox speciation has not been conclusively resolved.

Recombination is the scourge of phylogenetic studies, and since it is known that recombination has played a significant role in the evolution of at least some poxviruses [18]–[20], we decided to take a more detailed, and perhaps, primitive approach to examining the evolution of the smallpox genome.

## Materials and Methods

### Retrieval of Genome Sequences and Alignment

The genomes used for the construction of the MSA were: HSPV-MNR76, DQ792504; VACV-CVA, AM501482; VACV-WR, NC_006998; VACV-Lister, AY678276; VACV-Cop, M35027; RPXV-Utr, AY484669; CPXV-AUS_1999, HQ407377; CPXV-GRI, X94355; CPXV-FIN_2000_MAN, HQ420893; CPXV-GER_1980_EP4, HQ420895; CPXV-GER_2002_MKY, HQ420898; CPXV-GER91, DQ437593; CPXV-GER_1998_2, HQ420897; CPXV-GER_1990_2, HQ420896; CPXV-FRA_2001_Nancy, HQ420894; CPXV-NOR_1994_MAN, HQ420899; CPXV-BR, NC_003663; CPXV-UK2000_K2984, HQ420900; TATV-DAH68, NC_008291; CMLV-CMS, AY009089; VARV-GBR44_harv, DQ441444; MPXV-ZAR, NC_003310; ECTV-Mos, NC_004105. Sequences were aligned using MAFFT [21] and both ends of the genome were removed leaving a 98 kbp core region spanning the VACV-WR genome nt 37060–134689, from gene VACV-WR-050 (RhoA signal inhibitor, virus release protein) to VACV-WR-144 (RNA polymerase (RPO132). The sequences were then manually edited using Base-By-Base (BBB) [22], [23] to correct alignment errors.

### Modifications to Find Differences Tool in Base-By-Base

The Find Differences tool in BBB was used to compare groups of user-selected sequences in an MSA; it identifies those columns (nucleotide positions) in the MSA that satisfy the following conditions: nucleotides must be identical in all of the sequences in group 1 (All the same column), different from all sequences in group 2 (All different column), optionally, additional groups of either column (All the same or All different) can be added to the comparison.

The tolerance parameter was added to the BBB software to allow differences to be found when only 1, 2, or 3 genomes in a large alignment fail to satisfy the Find Differences search. For example, if VARV, CMLV and TATV have a unique block of sequence that is not present in 18/20 of the other genomes in the MSA then Find Differences will ignore the other 2/20 that have the unique block of sequence and print out the names of the “tolerated genomes” with the found positions. The tolerance parameter can be set for the all same or the all different group of viruses in these searches.

### Phylogenetic Tree Construction

A neighbor-joining (NJ) tree was constructed to perform phylogenetic analyses, as did Qin et al. [20]. All trees were constructed with MEGA5 [24] using the Tamura-Nei model and phylogenies were tested with 1000 bootstrap replicates; similar trees were also obtained using a maximum parsimony method (data not shown).

### Statistical Analyses

We tested the statistical significance of SNP clustering by binning the data and then calculating a p-value for the number of SNPs in each bin under a null hypothesis of a Poisson distribution. In order to ensure this result was robust to binning parameters we tested different bin sizes (250 nt, 500 nt, 750 nt, 1000 nt) as well as start positions (0 nt, +250 nt, +500nt), which had no effect on the significance of the peak we observe (data not shown). We also used a non-parametric method in which we randomly permute the SNPs throughout the region 1,000,000 times and record the bin with the largest number of SNPs and used this distribution to determine a significance cutoff; this gives us the same result as the parametric method (data not shown).

## Results

**Figure 1:**
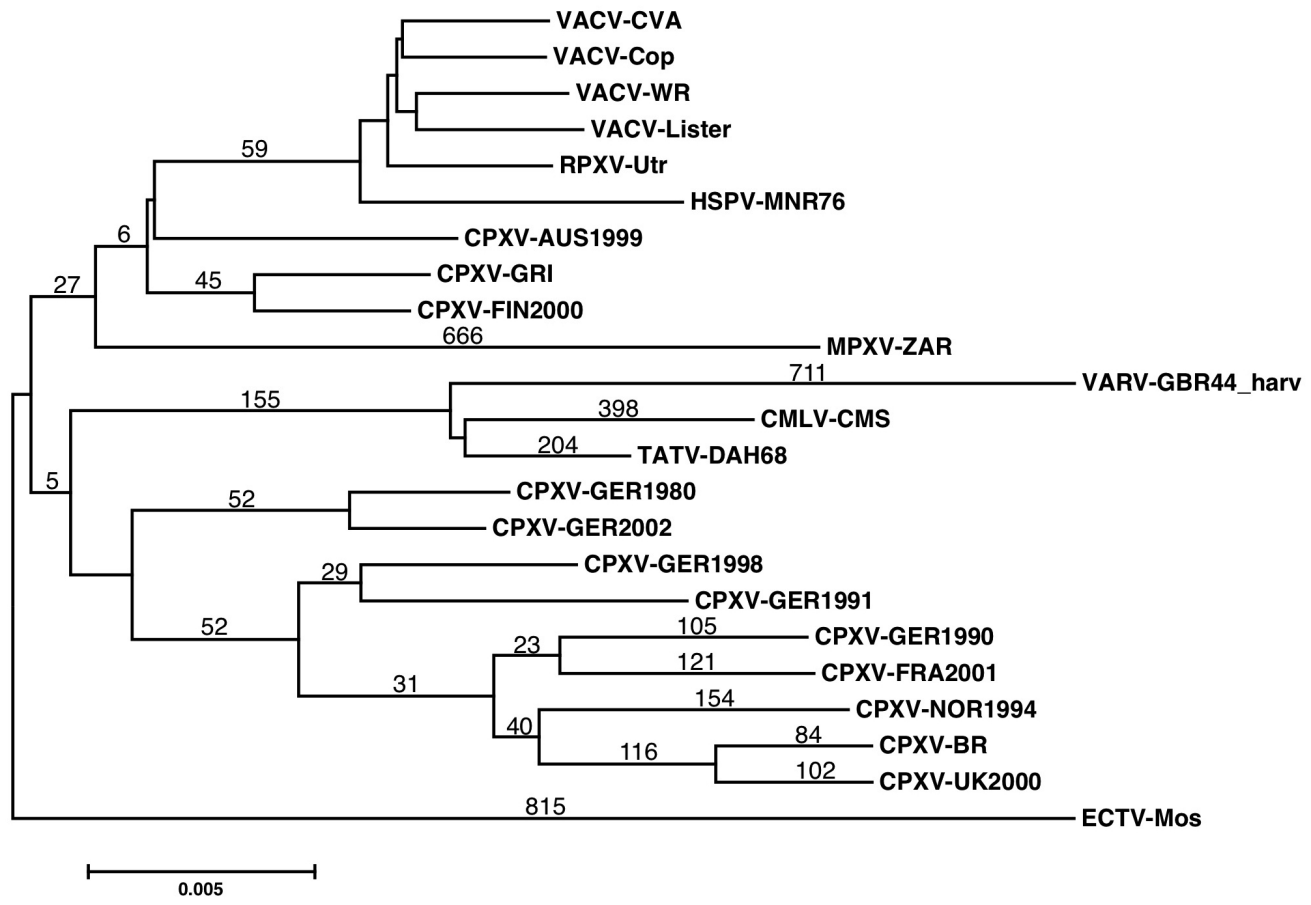
Neighbor joining tree created with MEGA5 using a 96 kbp MSA from the core of the genomes. Numbers on terminal branches indicate SNPs unique to individual viruses (gaps excluded). Numbers on internal branches indicate SNPs present in all viruses (gaps excluded) forming a particular clade and absent in all other viruses. Scale bar indicates nucleotide substitutions per site.

Individual VARV genomes have approximately 700 unique SNPs when compared to the common ancestor with CMLV and TATV, and when compared to individual CPXVs, the pairs of genomes have approximately 2–3,000 SNPs (ignoring gaps; in the core alignment used below) representing changes in both CPXV and VARV genomes from the most recent common ancestor (MRCA; data not shown); this number is too large to identify potential *smallpox-defining* changes. Therefore, since CMLV and TATV, which form a unique clade with VARV, also have very restricted host ranges, we decided to take a different approach and focus on the SNPs common to these three viruses and different in all other orthopoxvirus genomes within our test group. An important feature of this analysis is that it is not confounded by the SNPs arising post-speciation of VARV, CMLV and TATV. A multiple alignment of the genomes was constructed using MAFFT [1], [21] and manually corrected. Following the common practice in phylogenetic analyses, all nucleotide columns in the multiple sequence alignment (MSA) that contained a gap character were removed (gap assignment is prone to error) and then trimmed from the left and right ends to give a reliably aligned MSA core of 96,793 nt present in all of the genomes and spanning the region from F10L to A24R in vaccinia virus strain Copenhagen (VACV-Cop). Unfortunately, the terminal genome regions are too variable to be included in the following analysis. In this MSA, 88,557 positions were invariant, 7,743 positions contained 2 different nucleotides, 480 positions contained 3 different nucleotides and only 13 positions included all four nucleotides; the transition:transversion ratio was 3.0 (Text S1). Although the exact sets of SNPs discovered could be slightly affected by the choice of genomes used to construct the starting MSA, all SNP positions were subsequently verified with the other members of a viral species if they existed; thus, it is important to note that using any of the other >40 VARV genomes gave the same result (data not shown). In general, genomes were selected on the basis that they were similar to a consensus for a species and VACV-MVA genomes, which contain a series of large deletions resulting from “forced-evolution” – passage of the virus in chick embryo fibroblasts [2], [3], [25], were excluded; the most varied VACVs and all of the CPXVs were used because of their diversity. However, it was necessary to avoid including large numbers of closely related viruses because coincident, but isolate-specific, SNPs reduced the more general species/clade specific signals that we were searching for. A phylogenetic tree was determined with MEGA 5.0 [4], [24] using the neighbor-joining method (Figure 1); other methods gave very similar trees, all with high confidence boot strap values (>95) for all nodes except for some of the VACVs (data not shown).

**Figure 2:**
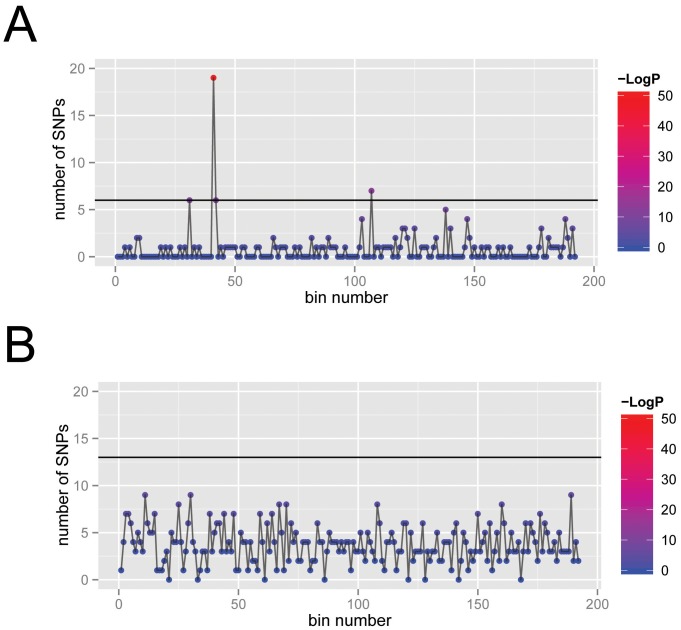
The 96 kbp MSA was split into 500 nucleotide bins and the number of SNPs in each bin are plotted. P-values for the number of SNPs in each bin are calculated from a null hypothesis of a Poisson distribution whose parameter is calculated using the average of all the bins; each bin is colored according to the negative log base 10 of the p-value. The horizontal line corresponds to a Bonferonni corrected p-value cutoff of 0.01. Panel A: SNPs unique to VARV, CMLV and TATV; Panel B: SNPs only found in VARV.

**Figure 3:**
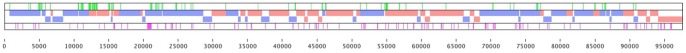
SNP distribution within the core region (96 kbp) of orthopoxvirus MSA. Green vertical bars indicate nucleotide positions removed from the MSA because of gaps. Vertical magenta bars indicate distribution of SNPs unique to VARV+CMLV+TATV genomes; pink and blue blocks indicate genes transcribed to the right and left, respectively. The block of magenta at approximately 21,000 nt is within the VACV-Cop O1L ortholog.

First we asked which nucleotide positions (SNPs) are identical in VARV+CMLV+TATV and different in **all** the other orthopoxvirus genomes in the alignment? This was initially performed looking at 1 genome of each species and subsequently confirmed with the other members. Such changes should have occurred during evolution from last common ancestor of the VARV+CMLV+TATV clade and the other orthopoxviruses to the separation of VARV and CMLV+TATV (Figure 1); a similar analysis with a subset of the CPXVs indicated the expected proportionality of branch length and number of SNPs across the orthopoxvirus groups (Figure 1). This analysis found 155 SNPs common to the core of the VARV, CMLV, TATV genomes. When the positions of these SNPs were plotted, it was apparent that these are distributed evenly though the genomes with the exception of one region of <1 kbp that has a significantly increased density of SNPs under the Poisson distribution (Figure 2A, methods). As a negative control we found no statistically enriched bins for SNPs unique to VARV (Figure 2B). In order to ensure the robustness of this enrichment we also tested a non-parametric method in which we randomly permuted SNPs through the MSA and used the region with the most SNPs as the background distribution; this method gave us the same result as above (data not shown). This cluster of SNPs is located within a 0.5 kbp region that maps to the 3′ half of the ortholog of VACV-O1L, a gene that is approximately 2.0 kbp (Figure 3; at approximately 21,000 in the core alignment). It was of special interest to note that VACV-O1L was recently discovered to be involved in VACV virulence and required for sustained activation of extracellular signal-regulated kinase (ERK)1/2 [5], [6], [26]. Although the absence of an O1L ortholog in VACV-MVA isolates, which have restricted host range [27], could be coincidental, it also points to a potential role for this gene in the control of host range, and thereby provided a tantalizing link to the speciation of VARV.

11 of the 26 SNPs, which clustered in this small region of the O1L ortholog, were non-synonymous and most of these generated conservative amino acid changes. These results are consistent with relatively subtle changes to protein function but indicate that most of the SNPs were not influenced by positive selection since no change in amino acid sequence occurs. Detailed evaluation of the MSA at the O1L locus, revealed a number of other SNPs present in each of the VARV, CMLV, TATV genomes, which were also present in one or two of the other genomes of the MSA and had therefore been missed by the original SNP search parameters. To allow more flexibility in the searches for the SNPs present in MSAs, the SNP counting software was recoded and a user-defined tolerance variable was incorporated; thus, the search became “Which SNPs are identical in VARV+CMLV+TATV and different in the other orthopoxvirus genomes **except 1, 2 or 3 tolerated genomes**?” The tolerances of 1 and 2 had a very pronounced effect on the spectrum of SNPs associated with the VARV+CMLV+TATV clade, increasing the number within the O1L ortholog by 14 and 28, respectively, with relatively little effect elsewhere in the genome. However, the effect was also remarkable for 1) the particular viruses that were matched to particular SNPs were almost exclusively CPXVs, and 2) the SNPs associated with the tolerated viruses clearly appeared as related groups. Examples include: 1) CPXV-GER1980 is the single tolerated virus in 6 of 7 SNPs within a 70 bp region, 2) CPXV-GER1980 and CPXV-AUS1999 are the two tolerated viruses over a 34 bp region with 8 SNPs and 3) for CPXV-NOR1994, 4 SNPs are tolerated over a 33 bp region, but 2 are also tolerated with CPXV-BR and the other 2 are tolerated with CPXV-FIN2000 (Table 1). Since the branches of the CPXV clades are strongly supported in phylogenetic analyses it was unexpected that, for the pairs of tolerated viruses, the individual CPXVs were not always siblings and, in fact, CPXV-GER1980 and CPXV-AUS1999 are now considered to be members of separate species [7], [8], [28]. Furthermore, these tolerated-SNPs also occur almost exclusively in the 3′ half of the O1L ortholog together with the SNPs unique to VARV, CMLV and TATV. Thus, to find all the SNPs associated with VARV, CMLV and TATV in this set of genomes, it was necessary to reduce the stringency of SNP detection and allow some tolerance of shared SNPs. It is also important to note that the selection of genomes used to build the MSA is critical because the inclusion of multiple almost identical sequences would greatly increase the tolerance values needed to pick out this second set of SNPs. These sets of tolerated SNPs, from different groups of CPXVs, together with the large number of synonymous SNPs suggest that recombination events were likely at play in the process of VARV evolution prior to the split from CMLV and TATV.

**Table 1:** SNPs common to VARV, CMLV, and TATV for tolerances 0, 1 and 2.

SNP Location		SNP Location (no gaps)	Tolerance	Tolerated Genome(s)	DNA Changes	AA Changes
225	*	224	0		CTC → CTA	
228		227	1	CPXV-GER91	CAA → CAG	
946		919	2	CPXV-FIN & NOR	ATA → TTA	I → L
957		930	2	CPXV-FIN & NOR	AGA → AGC	R → S
976978		949951	2	CPXV-BR & NOR	TCT → ACA	S → T
1116	*	1059	0		GTT → GTA	
1119	*	1062	0		GAG → GAT	E → D
1233	*	1176	0		TCT → TCC	
1248	*	1191	0		CTA → CTG	
1257	*	1197	0		ATC → ATA	
1263		1203	1	ECTV-Mos	GGA → GGT	
1290		1230	1	CPXV-NOR	ACC → ACA	
1299	*	1239	0		TCC → TCA	
1350	*	1290	0		GAG → GAT	E → D
1363		1303	1	ECTV-Mos	GCT → ACT	A → T
1383	*	1323	0		AGA → AGG	
1387	*	1327	0		CTG → GTG	L → V
1392		1332	1	CPXV-GER90	TCG → TCT	
1417	*	1357	0		GTA → ATA	V → I
1428	*	1368	0		GAT → GAC	
1432	*	1372	0		CAC → AAC	H → N
1437	*	1377	0		ACA → ACC	
1449	*	1389	0		TTA → TTG	
1454	*	1394	0		GAT → GCT	D → A
1458	*	1398	0		AGG → AGA	
1459	*	1399	0		CTA → TTA	
1493		1433	1	CPXV-GER91	AAA → AGA	K → R
1519		1459	1	CPXV-GER91	CGA → AGA	
1554	*	1494	0		AAC → AAT	
1558	*	1498	0		AAT → GAT	N → D
1568	*	1508	0		AAA → AGA	K → R
1578	*	1518	0		AAT → AAC	
1584	*	1524	0		TTA → TTG	
1638	*	1578	0		AAG → AAA	
1641	*	1581	0		ACG → ACA	
1650	*	1590	0		CCA → CCG	
1659	*	1599	1	CPXV-FIN	GAT → GAG	D → E
1695		1635	1	CPXV-GER80	TCT → TCC	
1698		1638	1	CPXV-GER80	GTG → GTA	
1725		1665	2	CPXV-GER80 & GRI	GTC → GTT	
17561758		16961698	1	CPXV-GER80	ATA → GTG	I → V
1761		1701	1	CPXV-GER80	GCT → GCC	
1764		1704	1	CPXV-GER80	ACT → ACC	
17981800		17381740	2	CPXV-GER80 & AUS	ACT → GCC → GTC	T → A → V
18021803		17421743	2	CPXV-GER80 & AUS	AAT → ACC → GCC	N → T → A
1804		1744	2	CPXV-GER80 & AUS	GTC → ATC	V → I
1808		1748	2	CPXV-GER80 & AUS	AAA → AGA	K → R
1815		1755	2	CPXV-GER80 & AUS	TTT → TTC	
1821		1761	2	CPXV-GER80 & AUS	TCC → TCA → TTA	No Change → S →L
1914		1854	2	CPXV-GER80 & GER91	GTG → GTA	

Each horizontal section represents a codon; occasionally 2 SNPs affect a single codon. When a second change is shown in the change columns, it refers to an additional VARV specific change in that codon, which must have happened after VARV diverged from CMLV and TATV. An asterisk indicates SNPs unique to VARV+CMLV+TATV.

**Figure 4:**
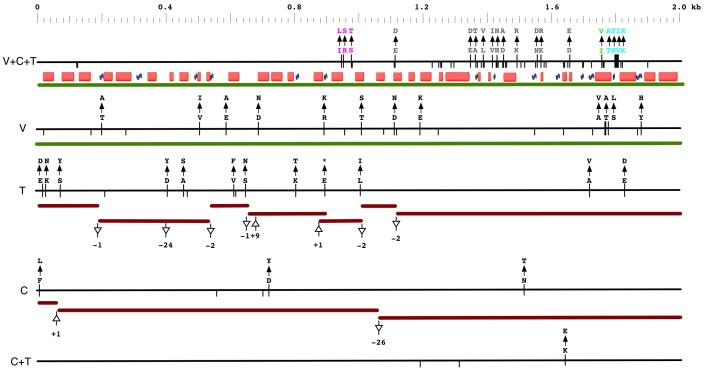
Distribution and consequences of SNPs in VARV, CMLV and TATV O1L orthologs. The DNA sequences of the O1L ortholog and pseudogenes are represented by horizontal black lines and labeled at the left side (V = VARV, C = CMLV, T = TATV). Tick marks below the lines indicate all SNPs, tick marks above the lines show non-synonymous SNPs with aa changes (arrow). V+C+T displays SNPs present in VARV, CMLV and TATV with a tolerance of 2; aa colored grey are unique to VARV+CMLV+TATV, other colors have been used to highlight aa changes associated with groups of more than 2 SNPs with similar sets of tolerated genomes (see Table 1). V, T, C and C+T lines display SNPs unique to VARV, CMLV, TATV and CMLV+TATV sequences, respectively; deletion (-ve values) and insertion (+ve values) of nucleotides are shown together with the fragmented ORFs (brown lines) created by frame-shifts. The green line represents the complete O1L orthologous genes and the consensus predicted secondary structure of the O1L proteins is shown as: red cylinder = alpha helix, blue = beta sheet.

The next series of analyses examine changes to O1L after the speciation of VARV, CMLV and TATV. The orthologous O1L gene is present in the CMLV and TATV genomes, but both viruses have accumulated multiple independent frame-shifting indels that disrupt the gene. Evaluation of the SNP distribution in this particular 96 kbp MSA revealed that only 11 SNPs were common to CMLV+TATV and different in all other genomes, and that the VARV, CMLV and TATV sequences had 711, 398 and 204 unique SNPs, respectively. An analysis of the O1L ortholog region revealed that after speciation: VARV accumulated 12 non-synonymous SNPs +11 synonymous SNPs; CMLV accumulated 3 non-synonymous SNPs +2 synonymous SNPs +2 independent indels; TATV accumulated 12 non-synonymous SNPs +3 synonymous SNPs +8 independent indels (Figure 4). The number, location and consequence of these post-split SNPs provide a window on the evolution of this gene. For VARV, which maintains the O1L gene, the post-split amino acid (aa) changes have a notable pattern: 4 are clustered around the 600 aa region of the predicted protein together with a block of pre-split aa changes, 8 are spread through the first 2/3 of the predicted protein and there is distinct absence of amino acid changes in the 430–580 aa region of the predicted protein where there is a very high concentration of aa changes resulting from the pre-split SNPs (Figure 4). Interestingly, there were 4 non-conservative aa switches (E>A, E>K, S>L and Y>H) among these post-split VARV changes. For TATV, the vast majority of post-split non-synonymous changes were in the 5′ half of the now pseudogene, and again there was a conspicuous absence of aa changes in the region equivalent to 430–580 aa of a predicted ortholog (Figure 4). The CMLV and TATV indels that generate ORF breaking frameshifts are within the first 10% of the O1L gene; therefore, these viruses are very unlikely to produce any type of functional polypeptide. These results suggest that there was a major change, creating multiple SNPs, to a relatively small region of the O1L ortholog in the last common ancestor of VARV, CMLV and TATV and that, subsequently, the gene was lost from both CMLV and TATV by independent mutations.

Searches of chordopoxvirus genomes for O1L orthologs showed that: 1) the gene is completely deleted from Yaba monkey tumor virus (YMTV; [9], [10], [29]); 2) three MYXV isolates [11], [12], [30], [31] have truncated genes (independent events), and 3) crocodilepox virus (CRV; [13], [14], [32]) was annotated with 3 copies of the O1L ortholog. YMTV, tanapox virus (TPV; [15], [33]) and yaba-like disease virus (YLDV; [16], [17], [34]) are poxviruses, which have limited host range and infect primates, including humans; YMTV produces a large localized tumor-like lesion, where as YLDV infections have been reported to resemble a mild form of smallpox. The fact that 3 of 23 MYXV genomes have truncated versions of the O1L ortholog is intriguing because 2 of these viruses are also described as very attenuated in wild rabbit populations [18]–[20], [31]. Of the 3 genes annotated in CRV (062, 063, 064), CRV-062 is the most likely to be a functional O1L ortholog. The other 2 are very dissimilar to each other, CRV-062 and to all other O1L orthologs (approximately 13–16% aa identity); protein sequence identity values this low place the matches deep in the *twilight-zone* of uncertainty. Indeed, the paralogous relationship of 062, 063 and 064 appears to be based solely on genome position, similar gene size and a very small patch of aa sequence similarity in the N-terminal region of the protein.

All similarity and motif searches failed to detect any non-poxvirus matches. However, it was interesting to note that the O1L protein orthologs are very diverse with percent aa identity values resembling those for the intracellular enveloped virus (IEV) protein F12L (VACV-COP; [21], [35]) rather than conserved core proteins (Table 2). However, the various O1L orthologs were all predicted to have similar, mostly alpha-helical, protein secondary structures (Figure 4). Although SignalP [22], [23], [36] and PrediSi [24], [37] do not predict a classical signal sequence at the N-terminus of O1L proteins, PSORT II [38], Jpred 3 [39] and TMHMM [40] consistently predicted a transmembrane domain at the C-terminus of the O1L proteins. The nuclear localization signal and leucine zipper motif (L.{6}L.{6}L.{6}L) noted previously for the VACV-Cop protein [26], were predicted inconsistently among the orthologous proteins; these motifs are relatively simple and tend to be over predicted.

**Table 2:** Amino acid identity values (%; gaps ignored) between VARV and other orthologs.

VARV-GBR44-Harv vs	O1L	J6R (RPO147)	F12L	A3L (P4b precursor)
CPXV-BR	94.7	99.1	95.6	98.5
ECTV-Mos	93.1	99.0	95.4	98.5
YKV-Dak	54.7	88.3	57.1	81.0
YLDV-Davis	38.6	80.8	36.0	66.4
DPV- W848	37.4	82.2	39.3	66.4
MYXV-Lau	34.5	81.5	37.1	63.4
COTV-SPA	30.8	75.3	36.0	66.3
MOCV	29.0	73.0	26.0	59.5

Calculated from pairs of sequences within a MUSCLE [48] multiple sequence alignment.

## Discussion

The SNP data analysis presented here indicates that an unusually high number of changes were introduced into the O1L ortholog after creation of the VARV+CMLV+TATV lineage but before VARV speciation. The origin of this gene is ancient, and although it is present in most members of all *Chordopoxvirinae* genera, including the North American orthopoxviruses (raccoonpox, skunkpox, volepox; I. Damon, personal communication) it is relatively poorly conserved compared to core proteins such as the RNA polymerase subunit RPO147 [41] or the virion core protein p4b [42] having a degree of conservation similar to the intracellular enveloped virion protein F12 [43] (Table 2). The distribution of the gene is especially curious. The loss of a functioning O1L gene from VACV-MVA and some MYXVs, both of which were artificially introduced into new host species, and from CMLV and TATV, which are natural pathogens with unique hosts, clearly shows that the protein is non-essential in some host/virus combinations; however, its general ubiquity also points to an important role in most viruses. Although deletion of the gene from VACV-CVA, the parent of VACV-MVA, has been shown to reduce plaque size and cytopathic effect in tissue culture and to attenuate the virus in mice [26], simple re-insertion of a functional O1L gene into VACV-MVA did not restore normal growth of the virus in human or mammalian cells [26]. All these results underscore the difficulties in dissecting host-range, replication and virulence phenotypes, which are influenced by multiple genes and external factors, such as host (natural or laboratory animal/tissue culture) or experimental model (infection route, outcomes measured). Furthermore, many of these answers also depend on the particular poxvirus in question because 1) these complex viruses encode multiple virulence genes, 2) the virulence genes are expressed at different levels by different viruses, and 3) multiple virulence proteins may function in the same pathway. Regarding the latter point, the VACV-O1L protein is involved in sustaining the virus-induced ERK1/2 activation, which is generated by the EGF-like viral growth factor (VGF) that in turn synergizes with the VACV-F1L ortholog to block apoptosis; deletion of either O1L or VGF can produce similar phenotypes [26], [44].

The data presented here supports previous hypotheses regarding the important role that recombination plays in the evolution of the poxviruses [18]–[20], [45]. Such recombination events may create relatively short, local inconsistencies in phylogenetic trees, which are not usually detectable by tools such as RDP2 [46] that have been designed for finding the types of gross sequence exchanges commonly found in such viruses as Human Immunodeficiency Virus and Hepatitis C Virus. Fully explaining the observed pattern of SNPs in the VARV OL1 gene is probably impossible, but the large block-like arrangement of these SNPs indicates a past recombination event with a sequence-donating virus that is not represented in the current databases. The events that created the smaller blocks of SNPs shared currently by VARV/CMLV/TATV and some of the CPXVs could have occurred before or after the transfer of the SNPs into the VARV/CMLV/TATV ancestor; some of the transfers could also be in either direction. Thus, early in the evolution of the VARV/CMLV/TATV lineage, the O1L gene had acquired a series of nucleotide differences that probably gave it unique qualities and may have altered its host range/virulence characteristics. Since most of the other approximately 700 SNPs that are unique to VARV seem to be unlinked, these results do not greatly effect the previous calculations of the dating of VARV’s emergence [2], [3], but they do suggest that if O1L is indeed a major player in VARV host range and virulence then these phenotypes may have undergone stepwise changes rather than a slow gradual modification. It is tempting to speculate that these changes in the O1L ortholog may be associated with both the gain of new host range and coincidental loss of others, but the actual separation of VARV, CMLV and TATV host ranges and the restriction to humans for VARV may be the result of subsequent SNP accumulation/gene loss. Although the function of O1L has not yet been determined beyond its role in sustaining ERK1/2 activation, it has also been noted that the protein contains HLA epitopes [47]; our evaluation of the VARV specific non-synonymous SNPs indicated that none of these were associated with the escape of a CTL response.

Finally, because the VARV O1L ortholog has been retained in all smallpox viruses and the host range of smallpox is strictly limited to humans, it is reasonable to assume that that the gene has undergone subsequent selection for optimal function in human cells and may in fact be important for the replication/infection cycle of VARV; however, how this relates to the virus case fatality rate cannot be predicted. Since deletion of the O1L gene from VACV-CVA only made the virus sensitive to drugs targeting the epidermal growth factor receptor (EGFR) in CV-1 (monkey) cells and not CEF cells [26], it is feasible that O1L or the ERK1/2 pathway may be good targets for anti-smallpox therapeutics. Similarly, because the VARV O1L ortholog may represent a humanized form of the gene, the expression of this protein in poxviruses used as vaccines or anti-cancer agents may be able to increase their efficacy by enhancing replication provided pathogenicity could be controlled by targeted deletion of other virulence genes such as those that directly target host immune functions.

## Supporting Information

Text S1 The file contains the multiple sequence alignment of genome cores, after gaps have been removed. The sequences are in FASTA format. The file can be loaded into the Base-By-Base software.
(FASTA)
